# Alox12/15 Deficiency Exacerbates, While Lipoxin A_4_ Ameliorates Hepatic Inflammation in Murine Alcoholic Hepatitis

**DOI:** 10.3389/fimmu.2020.01447

**Published:** 2020-07-14

**Authors:** Alexander Queck, Annika F. Fink, Evelyn Sirait-Fischer, Sabrina Rüschenbaum, Dominique Thomas, Ryan G. Snodgrass, Gerd Geisslinger, Hideo A. Baba, Jonel Trebicka, Stefan Zeuzem, Andreas Weigert, Christian M. Lange, Bernhard Brüne

**Affiliations:** ^1^Department of Internal Medicine 1, University Hospital, Goethe-University Frankfurt, Frankfurt, Germany; ^2^Faculty of Medicine, Institute of Biochemistry I, Goethe-University Frankfurt, Frankfurt, Germany; ^3^Department of Gastroenterology and Hepatology, University Hospital Essen, University of Duisburg-Essen, Essen, Germany; ^4^Institute of Clinical Pharmacology, Goethe-University Frankfurt, Frankfurt, Germany; ^5^Branch Translational Medicine, Fraunhofer Institute for Molecular Biology and Applied Ecology, Frankfurt, Germany; ^6^Department of Pathology, University Hospital Essen, University of Duisburg-Essen, Essen, Germany; ^7^European Foundation for the Study of Chronic Liver Failure, Barcelona, Spain; ^8^Institute of Clinical Research, Odense University Hospital, University of Southern Denmark, Odense, Denmark; ^9^Institute for Bioengineering of Catalonia, Barcelona, Spain

**Keywords:** alcoholic hepatitis, arachidonate 12/15-lipoxygenase (Alox12/15), specialized pro-resolving lipid mediators (SPMs), resolution of inflammation, lipoxin A_4_

## Abstract

Alcoholism is one of the leading and increasingly prevalent reasons of liver associated morbidity and mortality worldwide. Alcoholic hepatitis (AH) constitutes a severe disease with currently no satisfying treatment options. Lipoxin A_4_ (LXA_4_), a 15-lipoxygenase (ALOX15)-dependent lipid mediator involved in resolution of inflammation, showed promising pre-clinical results in the therapy of several inflammatory diseases. Since inflammation is a main driver of disease progression in alcoholic hepatitis, we investigated the impact of endogenous ALOX15-dependent lipid mediators and exogenously applied LXA_4_ on AH development. A mouse model for alcoholic steatohepatitis (NIAAA model) was tested in Alox12/15^+/+^ and Alox12/15^−/−^ mice, with or without supplementation of LXA_4_. Absence of Alox12/15 aggravated parameters of liver disease, increased hepatic immune cell infiltration in AH, and elevated systemic neutrophils as a marker for systemic inflammation. Interestingly, i.p. injections of LXA_4_ significantly lowered transaminase levels only in Alox12/15^−/−^ mice and reduced hepatic immune cell infiltration as well as systemic inflammatory cytokine expression in both genotypes, even though steatosis progressed. Thus, while LXA_4_ injection attenuated selected parameters of disease progression in Alox12/15^−/−^ mice, its beneficial impact on immunity was also apparent in Alox12/15^+/+^ mice. In conclusion, pro-resolving lipid mediators may be beneficial to reduce inflammation in alcoholic hepatitis.

## Introduction

Excessive alcohol consumption is one of the leading causes of liver associated morbidity and mortality worldwide (1). Contrary to the regressive prevalence of viral hepatitis, based on novel treatment options, the rate of alcoholic liver disease (ALD) is continuously increasing (2). ALD comprises different degrees of severity, from mild fatty liver disease to severe alcoholic hepatitis (AH) (3). Severe alcoholic steatohepatitis, either with or without the presence of liver cirrhosis, frequently presents itself as acute-on-chronic liver failure, a syndrome with high lethality (4). Currently, there is no satisfying treatment option for acute decompensation of alcoholic liver disease. Liver transplantation is an option only for highly selected patients and corticosteroids (5), as the currently only approved drug, showed only a minor and short-lived benefit compared to placebo (6, 7).

In the pathogenesis of AH, chronic alcohol consumption leads to fatty and fibrotic degeneration of the liver. Furthermore, enhanced local and systemic inflammation are main drivers of disease progression, namely of severe AH (8, 9). Already in steady state, continuous translocation of antigens and bacterial products like lipopolysaccharide from the gut via the portal vein to the liver are causatively involved (10, 11). Chronic alcohol consumption increases this dysfunction of the intestinal barrier, provoking hepatic and systemic inflammation (12). Moreover, immune dysfunction with consecutive reduced pathogen defense is a feature of AH (13). As a result, bacterial infections such as, e.g., spontaneous bacterial peritonitis, trigger acute decompensation of liver disease and heavily increase disease-related mortality (14, 15). Actively triggering resolution of chronic and acute inflammation during the development and decompensation of AH may therefore be of interest.

Resolution of inflammation serves to clear inflammatory cells and mediators and restores the endothelial barrier to regain tissue homeostasis (16, 17). During resolution, activity of immune cells (neutrophils, CD4^+^T_H1_- and T_H17_-cells, CD8^+^T-cells) is terminated by induction of apoptosis and their subsequent removal by efferocytosis (18). A group of specialized pro-resolving lipid mediators (SPMs: lipoxins, maresins, protectins, and resolvins) is thought to resolve inflammation without, in contrast to corticosteroids, suppressing essential properties of anti-microbial immunity such as T-cell activation (19). SPMs are produced by serial use of certain lipoxygenases (ALOX5, ALOX12, and ALOX15 in humans, Alox5 and Alox12/15 in mice) by predominantly oxygenating polyunsaturated fatty acids, i.e., arachidonic-, eicosapentanoic-, and docosahexanoic-acid. In the presence of these lipid mediators, cytokine production by activated CD8^+^T-cells and CD4^+^T-cells (T_H1_ and T_H17_) was significantly reduced, at least in animal models. Mice with a deficiency in docosahexaenoic acid synthesis and therefore impaired production of maresins and resolvins, showed significantly higher amounts of pro-inflammatory T_H1_- and T_H17_-cells and lower amounts of regulatory T-cells (19, 20).

Because of severe inflammation, impaired resolution of inflammation, and restricted bacterial clearance in alcoholic steatohepatitis, investigating pro-resolving lipid mediators as a treatment option was deemed promising. We hypothesized that lacking SPMs in ALOX12/15^−/−^ mice would aggravate alcoholic hepatitis compared to mice with intact SPM synthesis. Moreover, we asked if therapeutic application of LXA_4_, a well-characterized SPM with pro-resolving activity at least in mice, improves liver damage in Alox12/15^−/−^ and Alox12/15^+/+^ mice subjected to an established animal model of alcoholic hepatitis (21).

## Animal Experiments, Methods, and Statistics

### Mouse Model of Alcoholic Hepatitis

Alcoholic hepatitis was induced in C57BL/6-mice (Alox12/15^−/−^ and Alox12/15^+/+^; eight to twelve-week-old male mice with a body weight >20 g) using the chronic and binge feeding model established by Bertola et al. (21). Mice received a liquid diet [Lieber-DeCarli (LDC) shake and pour control liquid diet—Bio-Serv, product no. F1259SP] for acclimatization for 5 days, before they were randomly distributed into four groups; [1] Alox12/15^+/+^ ethanol and [2] Alox12/15^−/−^ ethanol, receiving Lieber-DeCarli ethanol liquid diet (5% Ethanol, Bio-Serv, product no. F1258SP), and [3] Alox12/15^+/+^ LDC and [4] ALOX12/15^−/−^ LDC, receiving Lieber-DeCarli control liquid diet. Liquid diet was given for another 11 days, with daily exchange and control of the nutriment. We used special liquid diet feeding tubes (Bio-Serv, product no. 9019, 50 ml) for better and comparable management of the diet supply. Ethanol diet was given *ad libitum*, whereas the amount of the control diet was pair-fed to ensure similar diet intake between the different groups. Weight and condition of the animals were daily controlled during the trial. After day 11, animals received an oral gavage with either diluted ethanol (5 g ethanol /kg BW) or diluted maltose dextrin (9 g maltose dextrin /kg BW) depending on the experimental groups (ethanol vs. LDC). After 9 h, animals were sacrificed, and liver and blood were obtained for further analysis. In a separate set of experiments, additive repetitive i.p. injections of LXA_4_ (LXA_4_; EMD Millipore, USA; 10 μg/kg bodyweight in 100 μl sodium chloride) were given at days 5, 7, 9, and 11 during chronic ethanol feeding. Animal experiments were performed in seven different cohorts to minimize influence of confounding factors. For animal experiments, the guidelines of the Hessian Animal Care and Use Committee were followed. The animal protocol was reviewed and accepted by the regional authority (application number: V54-19c20/15-FU/1193).

### Experimental Methods

#### AST/ALT-Measurement

Blood was collected in heparin tubes by direct puncture of the left heart ventricle. After centrifugation (2.000 × g for 10 min, 4°C), 32 μl blood plasma per measurement was analyzed using diagnostic test strips (Reflotron®, Roche, Germany, ALT and AST) and the Reflotron® Plus system, Roche, Germany.

#### Immunohistochemistry and Immunofluorescence Analysis of the Liver

For histochemistry, mice livers were perfused to remove blood and were fixed with a zinc fixative (100 mM Tris (pH 7.4) with 3.2 mM calcium acetate, 27.3 mM zinc acetate and 36.7 mM zinc chloride) for 6–8 h at 4°C. Afterwards, paraffin slides were created and underwent H&E staining. Images were created on an EVOS XL Core (Thermo Fisher) at 40× magnification. The software ImageJ was used to apply scale bars. Forty fields of a single H&E stained liver slice per time point were assessed by an expert liver pathologist to quantify hepatic steatosis, which was classified in grade 0–2 (i.e., 0%, ≤ 50% or >50% of hepatocytes are steatotic). Histological Sirius red staining and analysis for hepatic fibrosis quantification was performed as described previously (22). For immunofluorescence analysis of neutrophil infiltrates in the liver, sections were stained with anti-Ly6G antibody (Biolegend, 1A8), and Opal 650 reagent pack in the BOND-RX Multiplex IHC Stainer (Leica). Nuclei were counterstained with DAPI and slides were mounted with Fluoromount-G (SouthernBiotech). Slides were imaged at 20× using Vectra3 imaging system and analyzed using inForm 2.4.9 software (both from Akoya Biosciences).

#### Immunophenotyping of Liver and Blood Immune Cells

##### Immune cells of the liver

Before withdrawal of the liver, perfusion with sodium chloride solution (0.9%) via the left ventricle of the heart was performed, to discharge blood and immune cells from intrahepatic vessels. After harvesting the livers, hepatocytes were removed using liver dissociation kit (Miltenyi Biotec).

##### Blood immune cells

Following blood collection, blood cells were isolated by centrifugation (500 × g, 5 min, 4°C), followed by erythrocyte lysis.

Single cell suspensions were counted, and 10^6^ cells were resuspended in 50 μl staining buffer (PBS/BSA 0.5%) and blocked with 2 μl Fc-blocking Reagent per tube (Miltenyi Biotec) for 10 min on ice. Then, the antibody mix (see Table S1), containing 50 μl of Brilliant Violet Buffer (BD Biosciences) was added for 20 min at 4°C in the dark. After washing, cells were resuspended in 300 μl PBS/BSA 0.5% and acquired on a BD LSR II Fortessa flow cytometer. Frequencies of cell populations were analyzed using FlowJo V10 software (Tree star). The gating strategy to define immune cell populations is shown in Figures S2, S3. For correct gating, fluorescence minus one control were used.

#### Cytokine Measurement by Cytometric Bead Array (CBA)

For determination of certain blood cytokine levels, as described before (23), murine IL-1β, IL-4, IL-6, IL-8, IL-10, IL-12p70, IL-17, TNF-α, IFN-γ, and MCP-1 Cytometric Bead Array flex sets (BD Biosciences) were used. Samples were acquired with a BD LSR II Fortessa flow cytometer (BD Biosciences) and data were analyzed with BD Biosciences' FCAP software (V3.0).

#### Liquid Chromatography-Mass Spectrometry (LC-MS/MS) for Lipid Mediator Analysis of Hepatic and Systemic Blood Levels in Alcoholic Hepatitis

Eicosanoids [5(S)-HETE, 15(S)-HETE, and prostanoids] in the extracted samples (blood plasma and liver tissue) were analyzed using LC-MS/MS as described earlier (24, 25). The LC/MS-MS system comprised a 5500 QTrap mass spectrometer (Sciex, Darmstadt, Germany), an Agilent 1200 binary HPLC pump (Agilent Technologies), and an HTC Pal autosampler (Chromtech, Bad Camberg, Germany). Eicosanoid standards were obtained from Cayman Chemical. For detection of 5(S)-HETE and 15(S)-HETE, sample extraction was performed with liquid–liquid extraction using ethyl acetate. The organic phase was removed under a stream of nitrogen, and the residues were reconstituted in 50 μl methanol/water/BHT (50:50:10–4, v/v/v) prior to injection into the LC-MS/MS system. For chromatographic separation, a Gemini NX C18 column and precolumn were used (Phenomenex, Aschaffenburg, Germany). A linear gradient was employed at a flow rate of 0.5 ml/min with a total run time of 17.5 min. The mobile phases were (A) water/ammonia (100:0.01, v/v) and (B) acetonitrile/ammonia (100:0.01, v/v). Retention times of 5(S)-HETE and 15(S)-HETE were 8.31 and 6.97, respectively. Peak quantification was performed with Multiquant software version 3.0.2 (Sciex) employing the internal standard method (isotope dilution mass spectrometry). The ratios of analyte peak area and internal standard area (*y*-axis) were plotted against concentration (*x*-axis), and calibration curves were calculated by least square regression with 1/square concentration weighting. For detection of prostanoids, extracted samples were spiked with isotopically labeled internal standards (TXB_2_-d4, PGD_2_-d4, PGE_2_-d4), 100 μl EDTA solution (0.15M) and 600 μl ethyl acetate, to quantify levels of thromboxane B_2_, prostaglandin D_2_ and E_2_. Specimens were vortexed, and subsequently centrifuged at 20.000 × g for 15 min. The organic phase was removed, and the extraction repeated by the addition of 600 μl ethyl acetate. After combining the organic fractions, a sparing stream of nitrogen was used for evaporation at a temperature of 45°C. Reconstitution of the residues was performed by the addition of 50 μl acetonitrile/water/formic acid (20:80:0.0025, v/v/v) and transferred to glass vials. The chromatographic separation was carried out using a Synergi Hydro-RP column (150 × 2 mm, 4 μm particle size and 80 Å pore size; Phenomenex, Aschaffenburg, Germany). A gradient program was employed at a flow rate of 300 μl/min. Mobile phase A was water/formic acid (100:0.0025, v/v) and mobile phase B was acetonitrile/formic acid (100:0.0025, v/v). Separation of the analytes was performed under gradient conditions within 16 min. The injection volume was 10 μl and the gradient program started with 90% A for 1 min. The mobile phase A was decreased to 60% within 1 min. After 1 min biding, the mobile phase was further decreased to 50% within 1 min and another held for 2 min. Within 2 min, mobile phase A was further decreased to 10% and held for 1 min. In the space of 1 min, the initial conditions were restored. The column was re-equilibrated for 6 min. For mass spectrometric, parameters were set as follows: Source temperature 500°C, ion spray voltage −4,500 V, curtain gas 40 psi, nebulizer gas 40 psi, and turbo heater gas 60 psi. Both quadrupoles were running at unit resolution. Analyst Software 1.6.3 and Multiquant Software 3.0.2 (both Sciex, Darmstadt, Germany) were used for analysis and quantification. The following precursor-to-product ion transitions were used for quantification: m/z 369.2 → m/z 195.0 for TXB2, m/z 351.2 → m/z 233.3 for PGD2, and m/z 351.2 → m/z 315.0 for PGE2. Peak area of the corresponding internal standard was used for correction of the peak area of each analyte. Calibration curves were constructed using linear regression with 1/×2 weighting. The coefficient of correlation was at least 0.99. Variations in accuracy were less than fifteen percent over the whole range of calibration, except for the lowest limit of quantification, where a variation in accuracy of twenty percent was accepted.

#### Measurement of Hepatic Fpr2, Cmklr1, and Gpr18 mRNA Expression by qPCR

RNA from snap-frozen liver was isolated using the PeqGold protocol (Peqlab Biotechnologie). RNA was transcribed into cDNA using the Maxima First Strand cDNA Synthesis Kit (Thermo Fisher Scientific). Quantitative RT-PCR was performed using PowerUp SYBR Green Master Mix on a QuantStudio 5 Real-Time-PCR-System (Thermo Fisher Scientific). All primers were commercial QuantiTect primer assays from Qiagen.

### Statistical Analyses

Data were analyzed using GraphPad Prism 5.0 (GraphPad Software, San Diego, CA). *p*-values were calculated using unpaired *t*-test, or Mann–Whitney test. To check for normal distribution, Kolmogorov–Smirnov or D'Agostino and Pearson omnibus normality tests were performed. Parametric or nonparametric tests were applied accordingly. Asterisks indicate significant differences between experimental groups. (^*^p < 0.05, ^**^p < 0.01, ^***^p < 0.001). Different sample numbers between individual analyses are due to technical reasons, not due to data exclusion.

## Results

### Absence of Alox12/15 Increases Severity of AH Induced by Chronic and Binge Ethanol Feeding

**Figure 1:**
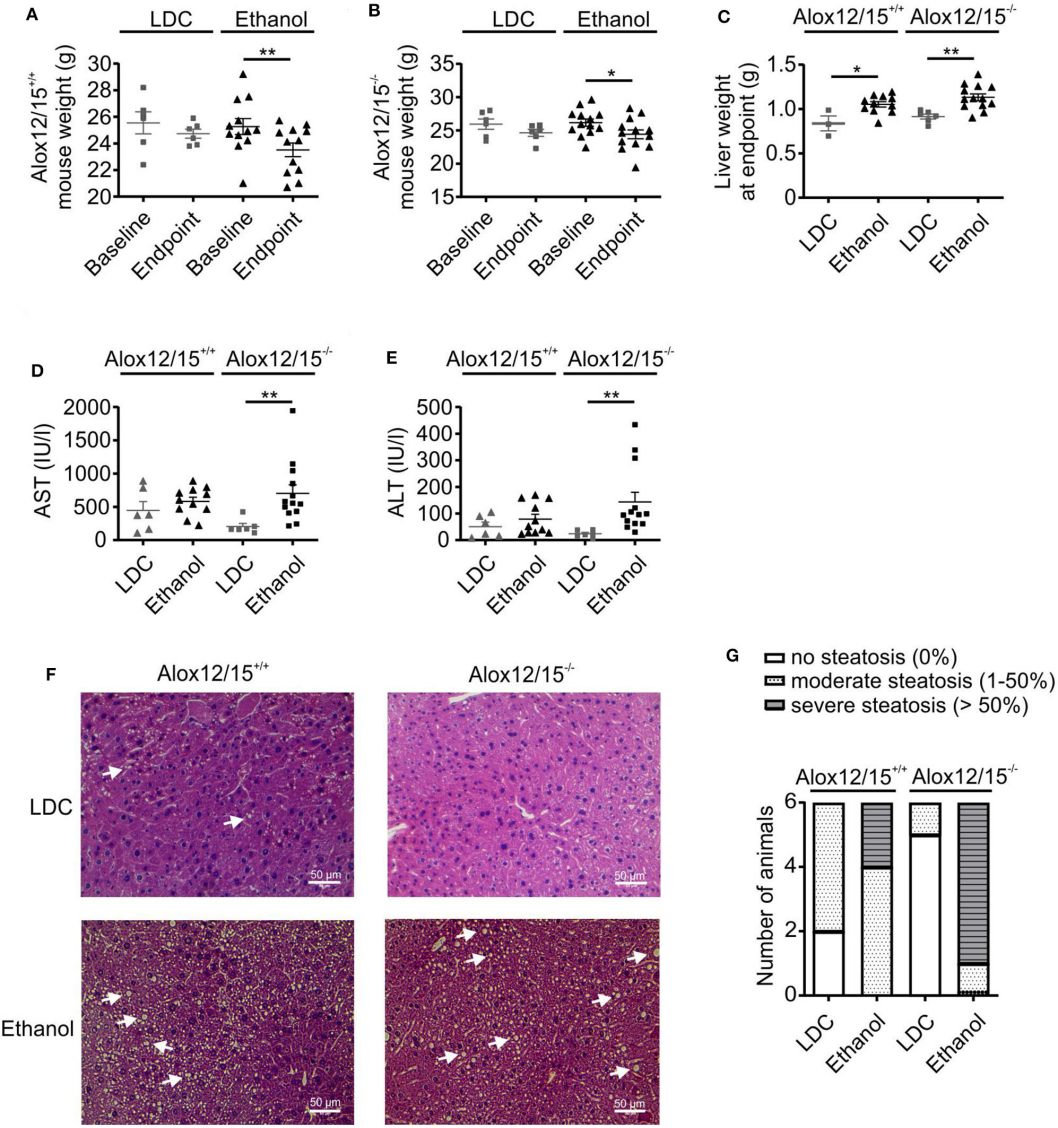
Alox12/15-deficiency increases markers of severity in AH. Alox12/15^+/+^ and Alox12/15^−/−^ mice were subjected to the NIAAA (National Institute of Alcoholism and Alcohol Abuse) model of alcoholic hepatitis (AH). After caloric acclimatization for 5 days, mice were fed with an ethanol containing diet for 11 days compared with an isocaloric liquid diet without ethanol (Lieber-DeCarli; LDC), followed by binge-feeding with ethanol or an isocaloric control. Body weight of Alox12/15^+/+^ **(A)** and Alox12/15^−/−^ **(B)** mice, liver weight **(C)**, as well as systemic aspartate aminotransferase (AST) **(D)** and alanine aminotransferase (ALT) **(E)** levels at the experimental endpoint are shown. Animal numbers were **(A,B)** Alox12/15^+/+^: LDC *N* = 6, Ethanol *N* = 12; Alox12/15^−/−^: LDC *N* = 6, Ethanol *N* = 13 **(C)** Alox12/15^+/+^: LDC *N* = 3, Ethanol *N* = 11; Alox12/15^−/−^: LDC *N* = 6, Ethanol *N* = 13 **(D,E)** Alox12/15^+/+^: LDC *N* = 6, Ethanol *N* = 11; Alox12/15^−/−^: LDC *N* = 6, Ethanol *N* = 13 **(F)** Representative H&E staining (magnification 40×) for detection of hepatic steatosis and inflammation in Alox12/15^+/+^ mice fed with LDC (top left) or Ethanol (bottom left) as well as Alox12/15^−/−^ mice fed with LDC (top right) or Ethanol (bottom right). Lipid droplets are indicated exemplarily with white arrows. **(G)** Grade of steatosis in Alox12/15^+/+^ and Alox12/15^−/−^ mice after LDC and ethanol feeding. **(F,G)** All groups *N* = 6. Wilcoxon matched-pairs signed rank test was used for comparison of LDC-, and paired *t*-test for comparison of Ethanol groups **(A,B)**. Mann–Whitney test was used for comparison between LDC-, unpaired *t*-test between Ethanol treated groups **(C–E)**. Each data point corresponds to data from one individual animal. *p ≤ 0.05, **p ≤ 0.01.

To analyze the impact of endogenous SPMs on AH development, Alox12/15^+/+^ and Alox12/15^−/−^ mice were subjected to the NIAAA model (National Institute of Alcoholism and Alcohol Abuse) (21). After caloric acclimatization, mice were fed with an ethanol containing diet for 11 days compared with an isocaloric liquid diet without ethanol (LDC), followed by binge-feeding with ethanol or an isocaloric control. Disease development was monitored using several parameters. Body weight, which was initially similar between experimental groups, increased during acclimatization to the liquid diet (5 days) (Figure S1A). Upon feeding with ethanol diet *ad libitum* or control diet pair-fed, mice continuously lost weight until the end of the experiment, indicating disease onset. However, mice showed a significantly lower body weight at the end of the experiment when they received ethanol (Alox12/15^+/+^ *P* < 0.01; Alox12/15^−/−^ *P* < 0.05) (Figures 1A,B and Figure S1A), which was independent of the genotype, although differences in daily intake between control and ethanol diet were not observed (Figure S1B). Liver weight was also significantly higher in both ethanol groups at the experimental endpoint, compared to the LDC groups, again largely being independent of the genotype (Alox12/15^+/+^ *P* < 0.05; Alox12/15^−/−^ *P* < 0.01; Figure 1C). Moreover, liver damage was assessed by monitoring AST and ALT levels in Alox12/15^+/+^ and Alox12/15^−/−^ mice at the experimental endpoint. Hereby, AST and ALT levels significantly increased in Alox12/15^−/−^ mice receiving ethanol compared to the LDC group, even though there was a trend in the WT mice as well (Figures 1D,E). Histological analysis revealed significant micro- and macro-vascular steatosis upon ethanol diet in both genotypes, while the LDC group only presented an infrequent moderate type of steatosis (Figure 1F). In detail, 33% of WT- and 83% of KO-mice presented severe steatosis (>50%) after ethanol exposure (Figure 1G). No relevant fibrosis was observed in all experimental groups (Figure S2).

### Altered Immune Cell Infiltrates Upon Alox12/15-Deficiency During AH

**Figure 2:**
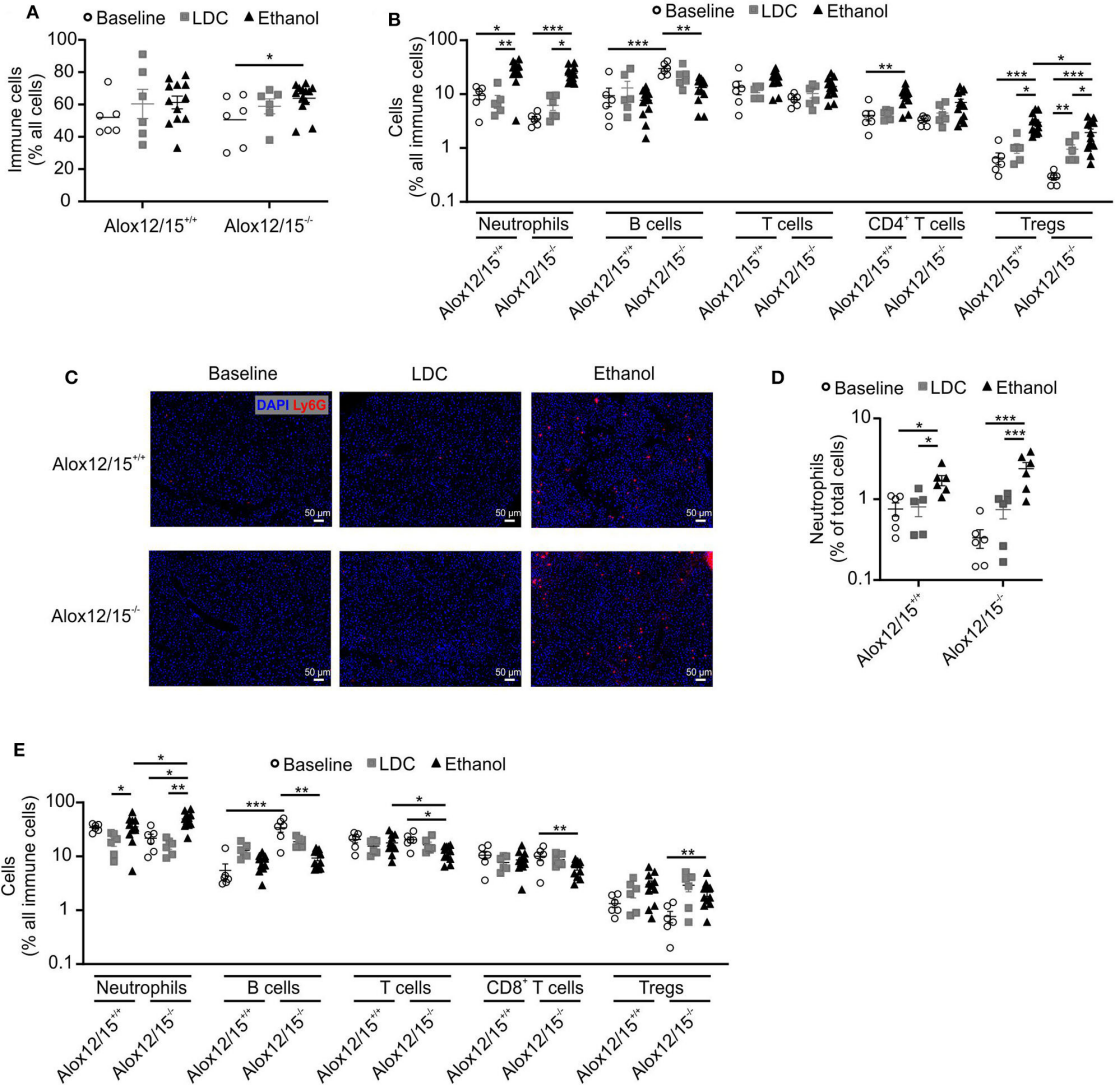
Impact of Alox12/15-deficiency on hepatic and systemic immune cells in AH. Alox12/15^+/+^ and Alox12/15^−/−^ mice were subjected to the NIAAA (National Institute of Alcoholism and Alcohol Abuse) model of alcoholic hepatitis (AH). Hepatic total immune cells **(A)** and immune cell subsets **(B)** are shown. **(C,D)** Representative images of hepatic neutrophil staining with Ly6G antibody **(C)** and the quantification of hepatic neutrophil levels **(D)** are shown. **(E)** Systemic immune cell subsets are shown. Animal numbers were: **(A–E)** Baseline, LDC *N* = 6 in both genotypes **(A,B)** Alox12/15^+/+^: Ethanol *N* = 12; Alox12/15^−/−^: Ethanol *N* = 13 **(C,D)** Ethanol *N* = 6 in both genotypes **(E)** Ethanol *N* = 11 in both genotypes. Mann–Whitney test was used for comparison between Baseline and LDC groups, unpaired *t*-test between Ethanol treated groups **(A,B,E)**. **(D)** Mann–Whitney test was used for comparison of all groups. Each data point corresponds to data from one individual animal. *p ≤ 0.05, **p ≤ 0.01, ***p ≤ 0.001.

To assess alterations in the immune response during AH, we performed FACS analysis of immune cell infiltrates in the liver at the experimental endpoint. We observed increased immune cell infiltration into the liver triggered by ethanol consumption (Figure 2A). While there was a tendency of increased immune cell infiltration in Alox12/15^+/+^ mice, we only observed a statistically significant change in Alox12/15^−/−^ mice, again indicating increased disease severity. To analyze alterations of individual immune cell subsets, we performed multispectral flow cytometry (gating strategies for liver and blood in Figures S3, S4). We found significantly elevated levels of hepatic neutrophils upon ethanol consumption compared to mice in the baseline (Alox12/15^+/+^ *P* < 0.05; Alox12/15^−/−^*P* < 0.001) and LDC groups (Alox12/15^+/+^ *P* < 0.001; Alox12/15^−/−^ *P* < 0.05; Figure 2B). This was confirmed by analyzing hepatic neutrophil infiltrates by immunohistochemistry (Ly6G). Significantly higher hepatic neutrophil levels were observed after ethanol treatment compared to LDC (Alox12/15^+/+^ *P* < 0.05; Alox12/15^−/−^*P* < 0.001) and untreated mice (Alox12/15^+/+^ *P* < 0.05; Alox12/15^−/−^*P* < 0.001), which was more pronounced in Alox12/15^−/−^ mice (Figures 2C,D).

Also, levels of blood neutrophils significantly increased upon ethanol consumption when compared to LDC-fed mice (both genotypes *P* < 0.05). Hereby, systemic levels of neutrophils were significantly higher in Alox12/15^−/−^ compared to Alox12/15^+/+^ mice (*P* < 0.05; Figure 2E). Regarding the adaptive immune system, a significant increase of hepatic CD4^+^ T-cells in Alox12/15^+/+^ animals was observed after ethanol consumption (*P* < 0.01). More specifically, CD4^+^ regulatory T-cells (T_reg_) were significantly elevated in the liver in Alox12/15^+/+^ and Alox12/15^−/−^ mice upon ethanol consumption (both *P* < 0.001), while significantly higher T_reg_ levels in Alox12/15^+/+^ compared to Alox12/15^−/−^ mice were detected (*P* < 0.05; Figure 2B). Systemic levels of T-cells decreased in both ethanol groups (significantly only in Alox12/15^−/−^ animals; *P* < 0.05) and were lower in Alox12/15^−/−^ than in Alox12/15^+/+^ animals (*P* < 0.05). Subset analyses revealed CD8^+^ T-cells as significantly decreased (*P* < 0.01) due to ethanol consumption in Alox12/15^−/−^ animals. In contrast, systemic T_reg_ levels significantly increased in Alox12/15^−/−^ animals triggered by ethanol consumption compared to animals at baseline (*P* < 0.01; Figure 2E). At baseline, hepatic B-cells were significantly higher in Alox12/15^−/−^ mice, compared to Alox12/15^+/+^ mice (*P* < 0.001). After ethanol or LDC treatment, however, this difference disappeared, because of a proportional higher reduction of B-cells in Alox12/15^−/−^ animals (*P* < 0.01; Figure 2B). The same scenario was evident at systemic B-cell levels. At baseline, B-cells were higher in Alox12/15^−/−^ animals (*P* < 0.001), with a significant reduction after ethanol treatment (*P* < 0.01; Figure 2E).

### Altered Cytokine and Lipid Mediator Production During AH

**Figure 3:**
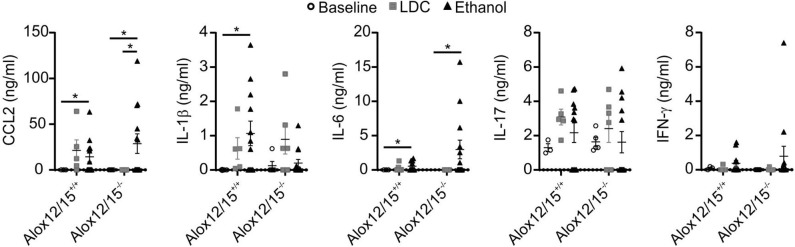
Impact of Alox12/15-deficiency on systemic cytokine levels in AH. Alox12/15^+/+^ and Alox12/15^−/−^ mice were subjected to the NIAAA (National Institute of Alcoholism and Alcohol Abuse) model of alcoholic hepatitis (AH). Systemic levels of the cytokine's chemokine (C-C motif) ligand 2 (CCL_2_), IL-1β, IL-6, IL-17, and IFN-γ are shown. Animal numbers were: Alox12/15^+/+^: Baseline *N* = 3, LDC *N* = 5, Ethanol *N* = 12; Alox12/15^−/−^: Baseline *N* = 5, LDC *N* = 6, Ethanol *N* = 13. For statistical analyses, Mann–Whitney test was used. Each data point corresponds to data from one individual animal. *p ≤ 0.05.

As expected, alcoholic hepatitis in mice altered inflammatory cytokine levels compared to healthy and LDC control mice. In both genotypes, blood levels of IL-6 and CCL_2_ were significantly elevated after ethanol consumption (both *P* < 0.05). Moreover, levels of IFN-γ and IL-17 were elevated after ethanol consumption, although statistical significance was not reached. Systemic levels of TNF-α, IL-8, IL-10, and IL-12p70 were below the detection limit of the assay. Importantly, Alox12/15^+/+^ mice showed a significant elevation of IL-1β levels during AH, which was not apparent in Alox12/15^−/−^ mice (*P* < 0.05). Even though comparison of cytokine levels after induction of AH between Alox12/15^+/+^ and Alox12/15^−/−^ mice showed no significant differences, levels of CCL_2_ and IL-6 showed a tendency to higher amounts in Alox12/15^−/−^ animals (CCL_2_: Alox12/15^+/+^ mean 14.3 ng/ml vs. Alox12/15^−/−^ mean 28.7 ng/ml; *P* = 0.5 and IL-6: Alox12/15^+/+^ mean 0.55 ng/ml vs. Alox12/15^−/−^ mean 2.98 ng/ml; *P* = 0.4). Contrarily, albeit also not significant, levels of IL-1β were higher in Alox12/15^+/+^ than in Alox12/15^−/−^ mice (IL-1β: Alox12/15^+/+^ 1 ng/ml vs. Alox12/15^−/−^ 0.2 ng/ml; *P* = 0.06; Figure 3).

To link AH development in Alox12/15^+/+^ mice vs. Alox12/15^−/−^ mice with changes in the production of SPMs or other inflammatory lipids, we profiled liver and blood samples for polyunsaturated fatty acid-derived lipid mediators. Importantly, there was no significant change in 5(S)-HETE and 15(S)-HETE, the primary arachidonic acid oxidation products of Alox5 and Alox12/15 between treatment groups and genotypes (Figure S5). Moreover, hepatic and systemic blood levels of prostaglandin D_2_ (PGD_2_), prostaglandin E_2_ (PGE_2_), and thromboxane B_2_ (TXB_2_) were detected after induction of AH in comparison to mice exposed to LDC diet. Particularly, hepatic levels of PGE_2_ significantly increased in Alox12/15^+/+^ mice subjected to the AH model (*P* < 0.05), and systemic levels of PGD_2_ and PGE_2_ increased in Alox12/15^−/−^ mice (*P* < 0.05; Figure S6). These findings did not allow to connect Alox12/15-deficiency with detectable SPM production, although an increase in systemic prostanoids may indicate shunting of arachidonic acid metabolism from Alox to cyclooxygenase products.

### Lipoxin A_4_ Attenuated Disease Pattern of Alcoholic Hepatitis, While Steatosis Progress

**Figure 4:**
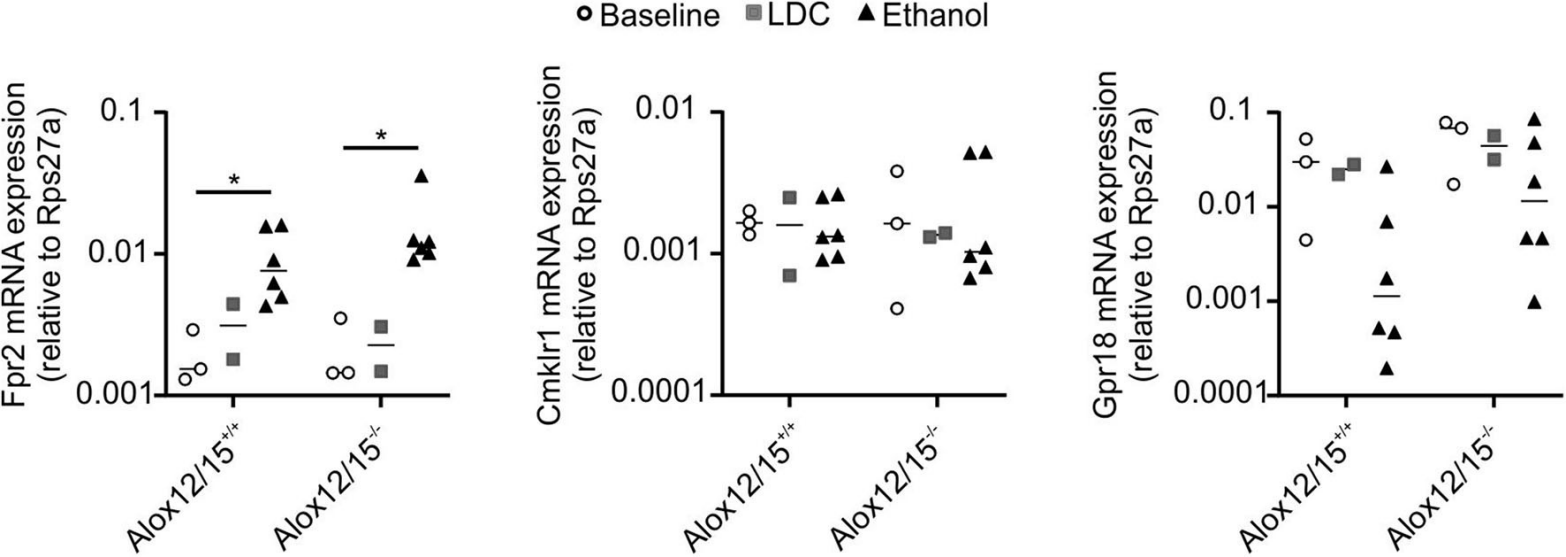
Impact of Alox12/15-deficiency and LDC/Ethanol diet on SPM receptor expression. Alox12/15^+/+^ and Alox12/15^−/−^ mice were subjected to the NIAAA (National Institute of Alcoholism and Alcohol Abuse) model of alcoholic hepatitis (AH). Expression of Fpr2, Cmklr1, and Gpr18 mRNA in snap-frozen liver tissue was determined by qPCR. Animal numbers were Alox12/15^+/+^: Baseline *N* = 3, LDC *N* = 2, Ethanol *N* = 6; Alox12/15^−/−^: Baseline *N* = 3, LDC *N* = 2, Ethanol *N* = 6. Mann–Whitney test was used for all statistical analyses. Each data point corresponds to data from one individual animal. *p ≤ 0.05.

**Figure 5:**
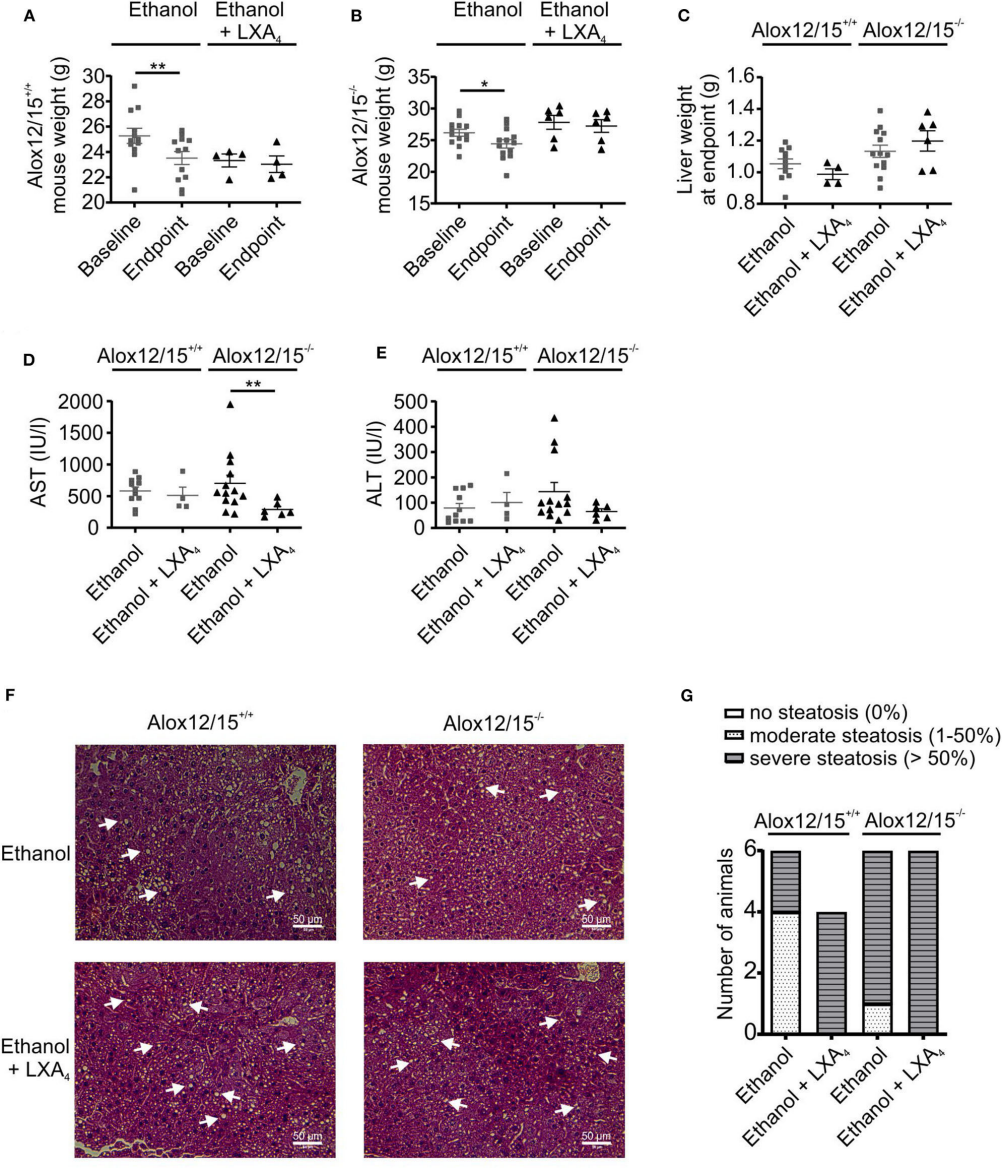
LXA_4_ improves disease parameters during AH. Alox12/15^+/+^ and Alox12/15^−/−^ mice were fed with an ethanol containing diet for 11 days followed by binge-feeding with ethanol, with or without repetitive i.p. injections of 10 μg/kg lipoxin A_4_ (LXA_4_) at days 5, 7, 9, and 11 during chronic ethanol feeding. Body weight of Alox12/15^+/+^ **(A)** and Alox12/15^−/−^ **(B)** mice, liver weight **(C)**, as well as systemic aspartate aminotransferase (AST) **(D)** and alanine aminotransferase (ALT) **(E)** levels at the experimental endpoint are shown. Animal numbers were: **(A)** Alox12/15^+/+^: Ethanol *N* = 12, Ethanol + LXA_4_ *N* = 4; **(B)** Alox12/15^−/−^: Ethanol *N* = 13, Ethanol + LXA_4_ *N* = 6; **(C–E)** Alox12/15^+/+^: Ethanol: *N* = 11, Ethanol + LXA_4_ *N* = 4; Alox12/15^−/−^: *N* = 13, Ethanol + LXA_4_ *N* = 6. **(F)** Representative H&E staining (magnification 40×) for detection of hepatic steatosis and inflammation in Alox12/15^+/+^ mice fed with ethanol without Lipoxin A_4_ i.p. injection (top left) and with Lipoxin A_4_ i.p. injection (bottom left) as well as Alox12/15^−/−^ mice fed with ethanol without Lipoxin A_4_ i.p. injection (top right) and with Lipoxin A_4_ i.p. injection (bottom right). Steatosis appears as white round structures, indicated by the white arrows. **(G)** Grade of steatosis in Alox12/15^+/+^ and Alox12/15^−/−^ mice after ethanol feeding with, or without LXA_4_ injections. Animal numbers were panels **(F,G)**. All groups *N* = 6, except for Alox12/15^+/+^: Ethanol + LXA_4_ *N* = 4. **(A,B)** Wilcoxon matched-pairs signed rank test was used for comparison of Ethanol + LXA_4_, and paired t test for comparison of Ethanol groups. **(C–E)** For statistical analyses of Ethanol + LXA_4_ groups Mann–Whitney test, and for Ethanol groups unpaired *t*-test was used. Each data point corresponds to data from one individual animal. *p ≤ 0.05, **p ≤ 0.01.

Since we were not able to directly measure SPMs, we wondered whether supplementation of a prototypical SPM might reverse changes observed in Alox12/15^−/−^ mice. This might serve as an indicator of changes in SPM production in these animals. Importantly, ethanol consumption increased hepatic mRNA expression of Lipoxin A_4_ receptor Fpr2, but not expression of Cmklr1 (receptor for Resolvin E_1_) and Gpr18 (receptor for Resolvin D_2_) (26, 27) (Figure 4). Therefore, LXA_4_ was chosen for supplementation in our model. Repetitive LXA_4_ injections were applied starting from day 5 after ethanol consumption. Upon LXA_4_ injection, body weight of animals of both genotypes stabilized and even increased for the last 2 days of the experiment, although no major difference in daily diet intake was observed, except on the last day of the experiment (Figure S7). When analyzed at the endpoint of the experiment, LXA_4_-treated animals did not lose a significant amount of weight over the whole experiment, which however was apparent in the ethanol group (*P* < 0.001; Figures 5A,B). In contrast, there was no impact on liver weight when LXA_4_ was applied during AH development (Figure 5C). When investigating markers of liver damage, significantly lower levels of AST were seen in LXA_4_-treated Alox12/15^−/−^ mice (*P* < 0.01), but not in Alox12/15^+/+^ mice (Figure 5D). Alox12/15^−/−^ mice also presented lower levels of ALT, although statistical significance was not reached (*P* = 0.15; Figure 5E). Thus, while mice of both genotypes benefitted from LXA_4_ treatment during AH, markers of liver damage where more affected in Alox12/15^−/−^ mice. Histological analysis revealed significant micro- and macro-vascular steatosis upon ethanol diet in combination with LXA_4_-injections in both genotypes (Figure 5F). All mice showed severe steatosis, compared to 33% of WT- and 83% of KO-mice after ethanol exposure without LXA_4_ injections (Figure 5G). No relevant fibrosis was present in all experimental groups (Figure S8).

### Lipoxin A_4_ Changed Immune Cell Infiltrates in Alcoholic Hepatitis

**Figure 6:**
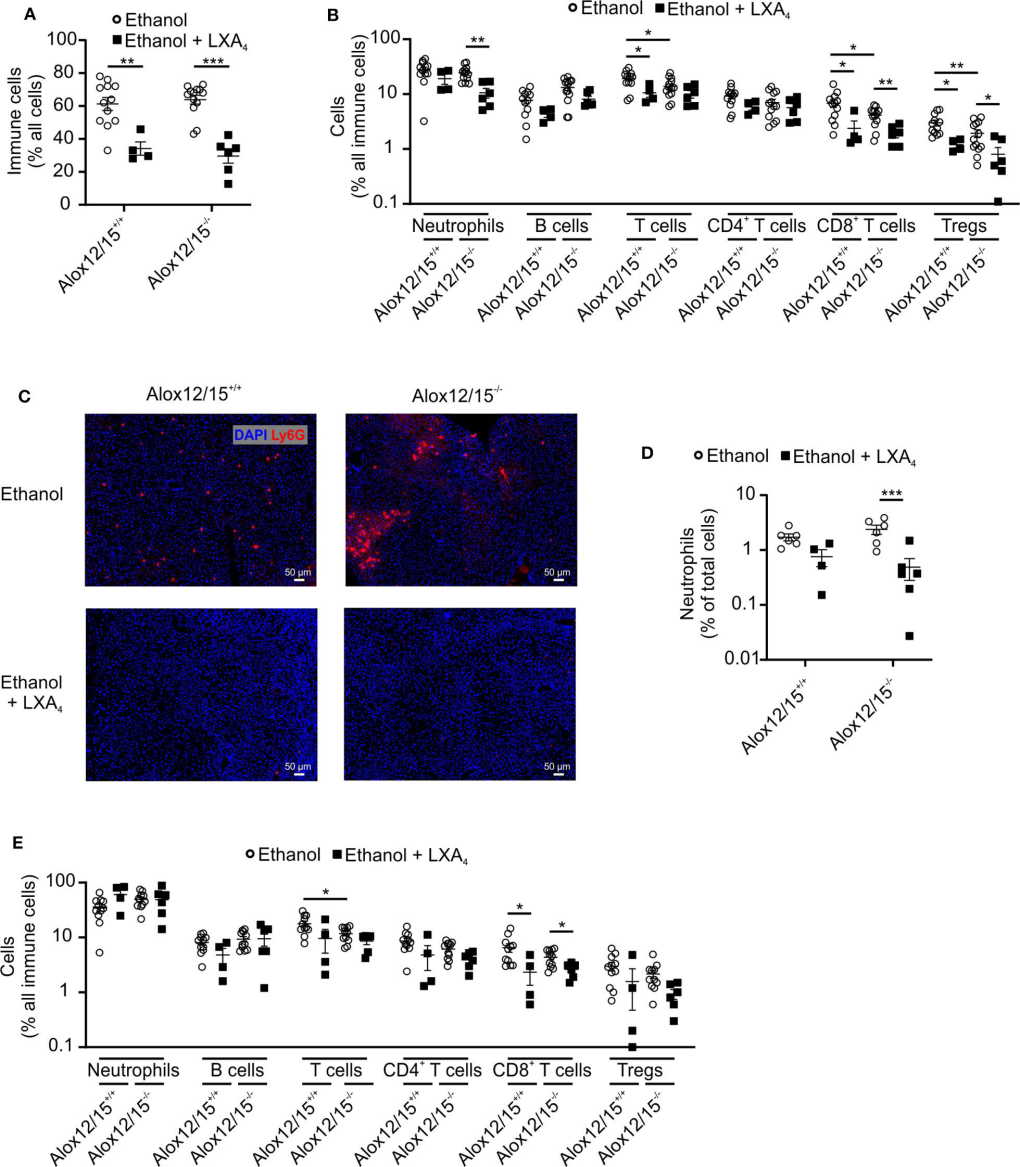
LXA_4_ decreases hepatic immune cell infiltrates during AH. Alox12/15^+/+^ and Alox12/15^−/−^ mice were fed with an ethanol-containing diet for 11 days followed by binge-feeding with ethanol, with or without repetitive i.p. injections of 10 μg/kg lipoxin A_4_ (LXA_4_) at days 5, 7, 9, and 11 during chronic ethanol feeding. Hepatic total immune cells **(A)** and immune cell subsets **(B)** are shown. Representative images of hepatic neutrophil staining with Ly6G antibody **(C)** and the quantification of hepatic neutrophil levels **(D)** are shown. **(E)** Systemic immune cell subsets are shown. Animal number were **(A,B)** Alox12/15^+/+^: Ethanol *N* = 12, Ethanol + LXA_4_ *N* = 4; Alox12/15^−/−^: Ethanol *N* = 13, Ethanol + LXA_4_ *N* = 6. **(C,D)** all groups *N* = 6. **(E)** Alox12/15^+/+^: Ethanol *N* = 11; Ethanol + LXA_4_ *N* = 4; Alox12/15^−/−^: Ethanol *N* = 11, Ethanol + LXA_4_ *N* = 6. For statistical analyses between groups with normal distribution (Ethanol) unpaired *t*-test was used. Mann–Whitney test was used for all other groups (Ethanol + LXA_4_) Each data point corresponds to data from one individual animal. *p ≤ 0.05, **p ≤ 0.01, ***p ≤ 0.001.

Taking body weight as an indicator of systemic inflammation, it was not surprising that LXA_4_-treatment altered immune cell dynamics in liver and blood. First, LXA_4_ injection significantly reduced ethanol-induced immune cell infiltration into the liver in both, Alox12/15^−/−^ and Alox12/15^+/+^ animals (*P* < 0.01; Figure 6A). Specifically, ethanol-associated infiltration of neutrophils into the liver decreased in Alox12/15^−/−^ and Alox12/15^+/+^ mice, although statistical significance was only reached in Alox12/15^−/−^ mice (*P* < 0.01). Immunohistochemical staining (Ly6G^+^) confirmed decrease of hepatic neutrophil count in both genotypes, also significant only in Alox12/15^−/−^ mice (*P* < 0.001; Figures 6C,D). Moreover, levels of hepatic T-cells (*P* < 0.05) were reduced in Alox12/15^+/+^ animals, with CD8^+^ T-cells being the mainly responsible subset (*P* < 0.05). However, levels of hepatic CD8^+^ T cells also dropped in Alox12/15^−/−^ mice (*P* < 0.01) after LXA_4_ injections. Furthermore, reduction of hepatic regulatory T-cells was seen in both genotypes (Alox12/15^+/+^ *P* < 0.01 and Alox12/15^−/−^ *P* < 0.05) (Figure 6B). Finally, at the systemic level only CD8^+^ T-cells were reduced upon LXA_4_-treatment in both genotypes (both *P* < 0.05; Figure 6E).

### Lipoxin A_4_ Reduced Pro-inflammatory Cytokines in Alcoholic Hepatitis

**Figure 7:**
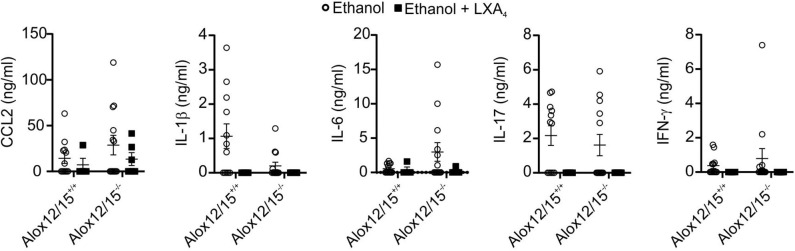
LXA_4_ decreases systemic cytokine levels during AH. Alox12/15^+/+^ and Alox12/15^−/−^ mice were fed with an ethanol containing diet for 11 days followed by binge-feeding with ethanol, with or without repetitive i.p. injections of 10 μg/kg lipoxin A_4_ (LXA_4_) at days 5, 7, 9, and 11 during chronic ethanol feeding. Animal numbers were Alox12/15^+/+^: Ethanol *N* = 12, Ethanol + LXA_4_ *N* = 4; Alox12/15^−/−^: Ethanol *N* = 13, Ethanol + LXA_4_ *N* = 6. For statistical analyses, Mann–Whitney test was used. Systemic levels of the cytokine's chemokine (C-C motif) ligand 2 (CCL_2_), IL-1β, IL-6, IL-17, and IFN-γ are shown. Each data point corresponds to data from one individual animal.

Corresponding to reduced inflammatory cell infiltrates in the liver, systemic levels of IL-1β, IL-6, IL-17, TNF-α, and IFN-γ were suppressed below the detection limit of the assay after LXA_4_-injections during AH development. Furthermore, levels of CCL_2_ decreased upon LXA_4_-injection compared to the ethanol group without LXA_4_-injections, although not statistically significant (Figure 7). In contrast to cytokines, blood and liver levels of 5(S)-HETE and 15(S)-HETE were not altered by LXA_4_ injection (Figures S5E,F).

## Discussion

Investigation of new treatment options in alcoholic liver disease (ALD) are urgently needed, while a mouse model to mimic non-fibrotic alcoholic liver disease exists (NIAAA model) (21). In the present study, this experimental protocol induced the development of microvascular fatty liver degeneration, increased hepatic transaminases, and hepatic immune cell infiltration as cardinal features of alcoholic liver disease (28). Besides direct toxicity of ethanol and ethanol degradation products such as acetaldehyde, hepatic, and systemic inflammation are the main driver in alcoholic liver disease progression. Hereby, chronic ethanol consumption features sterile and nonsterile inflammatory pathways. Bacterial translocation from the gut, due to impairment of the intestinal barrier by ethanol (29) leads to recognition of pathogen associated molecular patterns (PAMPs) e.g., by toll-like receptors. Additionally, ethanol provokes the release of damage-associated molecular patterns (DAMPs), e.g., by liver cells undergoing cell death (8). For prevention of parenchymal damage and return to homeostasis, this pro-inflammatory signaling needs to be terminated after pathogen clearance (18). During resolution of inflammation pro-resolving mediators such as LXA_4_ stop chemotaxis, adherence and transmigration of neutrophils (30), stimulate non-phlogistic phagocytosis of apoptotic cells by macrophages (31) and inhibit TNF-α secretion by T-cells by blocking ERK activation (32). Our hypothesis was that lack of pro-resolving lipid mediators in Alox12/15^−/−^ mice would prohibit resolution of hepatic inflammation and support pro-inflammatory signaling by systemic and hepatic inflammatory immune cells (33). Indeed, our data reveal higher levels of systemic inflammation, suggested by elevated blood neutrophils when Alox12/15 was absent. However, other signs of disease severity and the levels of hepatic neutrophils were comparably elevated in both genotypes. Despite being required for SPM generation, Alox12/15 is also involved in production of other lipid metabolites (34). Hereby, depending on the substrate, hydroxyoctadecadienoic acids (HODEs) from linoleic acid and hydroxyeicosatetraenoic acids (HETEs) from arachidonic acid are generated. Recently, Zhang et al. reported enhanced liver damage based on increased levels of 13-HODE in mice subjected to an AH model. Accumulation of 13-HODE increased reactive oxygen species, apoptosis, and alterations in lipid metabolism, while genetic depletion of Alox12/15^−/−^ ameliorated this damage (35). Consequently, analyzing the impact of pro-resolving mediators on hepatic damage in alcoholic steatohepatitis using Alox12/15 knockout is influenced by the generation of HODEs, due to a linoleic acid-enriched diet in the NIAAA model. This could have suppressed the benefit of enhanced resolution of inflammation in WT animals and could have prevented presentation of an even more beneficial phenotype in our experiments. Unfortunately, we were not able to assess SPM production in our model, since SPM levels were all below the detection limit in our LC-MS/MS analyses in both blood and liver. This is not uncommon in the field, and may at least be partly attributed to low stability of SPMs (36, 37). We therefore asked if changes in precursor molecules might be detectable. When considering arachidonic acid metabolites these, at least in humans, are 5-HETE and 15-HETE. We did, however, not detect changes in 5-HETE and 15-HETE levels upon mouse Alox12/15 ablation. Again, this phenomenon was demonstrated before in the literature and may be due to arachidonic acid oxidation in these positions by other enzymatic and particularly non-enzymatic sources (36). Absence of changes in precursor molecules might also be due to the relatively low amounts of precursors that are finally converted into SPMs, which requires a transcellular mechanism (38). While issues cannot indicate that endogenous SPMs are irrelevant in our model, other approaches should be used in the future to study the impact of endogenous SPMs, such as ablation or antagonism of SPM receptors.

Not only changes in neutrophils were triggered by the induction of AH in Alox12/15^−/−^ vs. Alox12/15^+/+^ animals. Also, significantly lower levels of hepatic regulatory T cells were detected in Alox12/15^−/−^ mice. Even if the role of regulatory T cells in ALD is still under investigation, their immunosuppressive competence through inhibition of antigen presenting cells and adaptive immune cells, e.g., by secretion of anti-inflammatory cytokines is well-established. Hence, a protective role in ALD is discussed, limiting inflammation and lipid accumulation via IL-10-dependent suppression of hepatic pro-inflammatory macrophages (39). Importantly, T_reg_ induction was identified as one feature of SPMs (20). This observation fits to the decreased hepatic T_reg_ frequency in Alox12/15^−/−^ mice.

SPMs use different receptors for anti-inflammatory signaling. LXA_4_, e.g., is known to interact with the G-protein coupled formyl peptide receptor 2 (Fpr2). Fpr2 is expressed on many cell types including hepatocytes, monocytes/macrophages, neutrophils, and endothelial cells, but its expression is particularly high on neutrophils (27). Importantly, hepatic Fpr2 mRNA expression in alcoholic hepatitis was increased, contrary to receptors used by other SPMs such as resolvins. This may reflect the increase in neutrophil influx upon ethanol diet. Hence, for proof of concept, repetitive intraperitoneal injections of LXA_4_ were used to induce resolution of inflammation in acute steatohepatitis. Interestingly, reduced hepatic immune cell infiltration, reduced systemic pro-inflammatory cytokine levels, and finally, recovery of mouse weight at the end of the experiment was seen with LXA_4_. In detail, levels of hepatic neutrophils were significantly reduced in mice after LXA_4_ injection, as were the levels of cytotoxic CD8^+^ T cells both in the liver and in peripheral blood. Several animal models already indicated the existence of T-cell mediated hepatitis because of alcohol consumption. Therefore, hepatic T-cell reduction might be a therapeutic treatment option in absence of bacterial infection (40, 41). Particularly, animals lacking Alox12/15 benefitted from LXA_4_-treatment, indicating that indeed a lack of SPMs may have been counterbalanced by LXA_4_ treatment. This was observed mainly as a significant reduction of AST and normalization of ALT in contrast to the ethanol group. Interestingly, these findings contrast with higher rates of severe steatosis in both genotypes after LXA_4_ injections. Aggravated steatosis, while markers of inflammation improved in the development of experimental AH, is a particularly noteworthy finding. While LXA_4_ is known to be mainly involved in the mechanism of resolution of inflammation, knowledge about the influence on lipid metabolism is relatively scarce. However, Börgeson et al. (42) reported additional reduction of hepatic triglyceride accumulation and hepatic steatosis in a mouse model of obesity-induced adipose inflammation through LXA_4_ injections. One explanation of steatosis progression in our model in response to LXA_4_ injections might be the improvement of the general condition of the animals, leading to increase of daily diet intake at the end of the experiment and stabilization of body weight, while mice in the ethanol group continuously lost weight over time. Another explanation might be that recruited immune cells play a role in lipid clearance, which would fit the concept of inflammation as a reaction triggered to clear mediators that disturb or endanger tissue homeostasis. However, further investigation of LXA_4_ downstream signaling in alcoholic liver disease is necessary for a more precise understanding of the cellular and molecular mechanisms how LXA_4_ affects the development of steatosis.

In conclusion, repetitive LXA_4_ injections improved hepatic inflammation in murine alcoholic hepatitis. Especially in mice putatively lacking pro-resolving lipid mediators, LXA_4_ reduced markers of inflammation and liver damage inflammation in alcoholic hepatitis. Further investigation of LXA_4_ and its downstream signaling pathways in alcoholic liver disease appear promising to validate SPM's as part of the therapeutic regimen to overcome AH-related mortality.

## Data Availability Statement

The raw data supporting the conclusions of this article will be made available by the authors, without undue reservation.

## Ethics Statement

The animal study was reviewed and approved by Regierungspräsidium Darmstadt, Dezernat V54, Veterinärwesen und Verbraucherschutz, Wilhelminenstraße 1-3, Wilhelminenhaus 64283 Darmstadt. Application number: V54-19c20/15-FU/1193).

## Author Contributions

AQ, AW, CL, and BB contributed to the manuscript by planning and initiating the study. AQ, AF, ES-F, SR, DT, GG, HB, AW, CL, and BB collected the data. AQ, AW, CL, and BB performed the statistics. AQ, AF, ES-F, RS, JT, SZ, AW, CL, and BB interpretated data. AQ, AW, CL, and BB drafted the manuscript. Funding acquisition: AQ, AW, CL, and BB. All authors critically discussed, corrected, and reviewed the manuscript.

## Conflict of Interest

The authors declare that the research was conducted in the absence of any commercial or financial relationships that could be construed as a potential conflict of interest.
