# Supplementary material for: Alox12/15 Deficiency Exacerbates, While Lipoxin A4 Ameliorates Hepatic Inflammation in Murine Alcoholic Hepatitis
Source: Front Immunol. 2020 Jul 14;11:1447. doi: 10.3389/fimmu.2020.01447 (PMC7371948; doi:10.3389/fimmu.2020.01447)
Supplement: Supplementary file 1 [file Data_Sheet_1.docx]

Supplementary Material

# Supplementary Figures and Tables

## Supplementary Figures

**Figure S1**

**
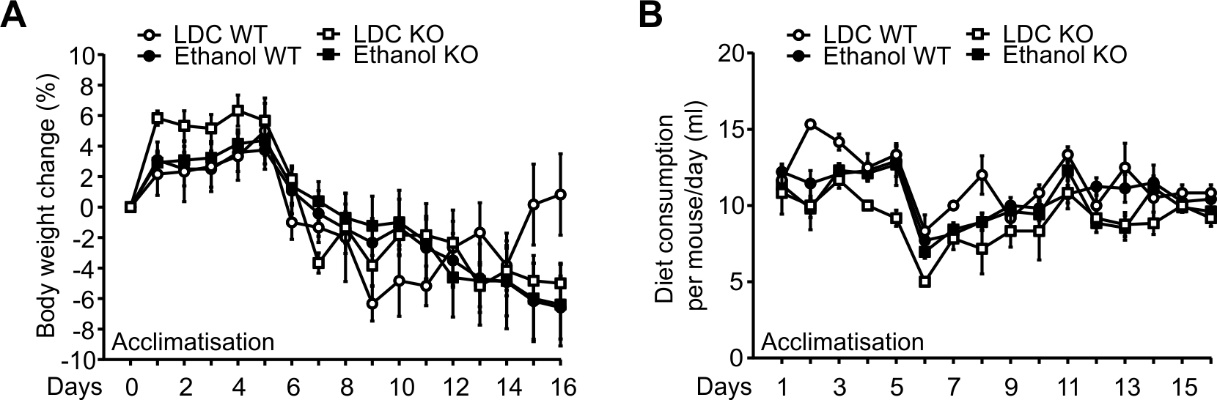
**

Figure S1: Body weight and diet intake during AH. Alox12/15^+/+^ and Alox12/15^-/-^ mice were subjected to the NIAAA (National Institute of Alcoholism and Alcohol Abuse) model of alcoholic hepatitis (AH). After caloric acclimatization for five days, mice were fed with an ethanol containing diet for 11 d compared with an isocaloric liquid diet without ethanol (Lieber-DeCarli; LDC), followed by binge-feeding with ethanol or an isocaloric control. (A) Change in body weight of Alox12/15^+/+^ (WT) and Alox12/15^-/-^ (KO) mice over time, and (B) diet intake over time are shown. Animal numbers were Alox12/15^+/+^: LDC N=6, Ethanol N=12; Alox12/15^-/-^: LDC N=6, Ethanol N=13. Data are means ± SEM.

**Figure S2**

**Figure S2. Representative Sirius Red staining (magnification 40X) for detection of hepatic fibrosis in AH.** Alox12/15^+/+^ mice fed with normal chow (top left), with LDC (top center) or Ethanol (top right), as well as Alox12/15^-/-^ mice fed with normal chow (bottom left), with LDC (bottom center) or Ethanol (bottom right). Representative Sirius Red staining (magnification 40X) for detection of fibrosis (appears as red structures) is shown.

**Figure S3**

#

Figure S3: Gating strategy for hepatic immune cells. After single cell identification, immune cells were separated by CD45 staining. CD11b versus CD19/Ly6G staining separates immune cells into lymphocytes (CD11b^-^), neutrophils (CD11b^+^ Ly6G^+^), and other CD11b^+^ cells. Lymphocytes were further divided into T cells (CD3^+^) and others. CD4^+^ and CD8^+^ T cells were identified by their respective lineage markers, while regulatory T cells in CD4^+^ T cells were detected using GITR antibody. CD8^+^ memory cells in CD8^+^ T cells were identified with Ly6c antibody. Double negative T cells (CD4^-^ and CD8^-^) contain γδT-, γδNK T- and NK T cells (separation via γδTCR versus NK1.1). Non-T lymphocytes contain B cells (MHCII^+^ CD11c^-^) and DCs (MHCII^hi^ CD11c^+^), and MHCII- NK1.1^+^ NK cells. CD11b^+^ cells included eosinophilic granulocytes, Ly6c^+^ and Ly6c^-^ monocytes, CD11b^+^ DCs, Kupffer cells and monocyte-derived macrophages by comparing MHCII versus MerTK/F4/80 expression, followed by either Ly6C versus SSC-A, MHCII versus CD11c, or CD11b versus MerTK/F4/80 comparison.

**Figure S4**

Figure S4: Gating strategy for blood immune cells. After single cell identification, immune cells were separated by CD45 staining. CD11b versus CD19/Ly6G staining separates immune cells into lymphocytes (CD11b^-^), neutrophils (CD11b^+^ Ly6G^+^), and other CD11b^+^ cells. Lymphocytes were further divided into T cells (CD3^+^), B cells (CD19^+^), and others. CD4^+^ and CD8^+^ T cells were identified by their respective lineage markers, while regulatory T cells in CD4^+^ T cells were detected using GITR antibody. γδT cells were identified in double negative T cells by expression of the γδTCR. Non-T and B lymphocytes contained NK1.1^+^ NK cells. CD11b^+^ cells (monocyte gate) contained eosinophilic granulocytes, Ly6c^+^ and Ly6c^-^ monocytes. Blood DCs were MHCII^hi^ CD11c^+^.

**Figure S5**


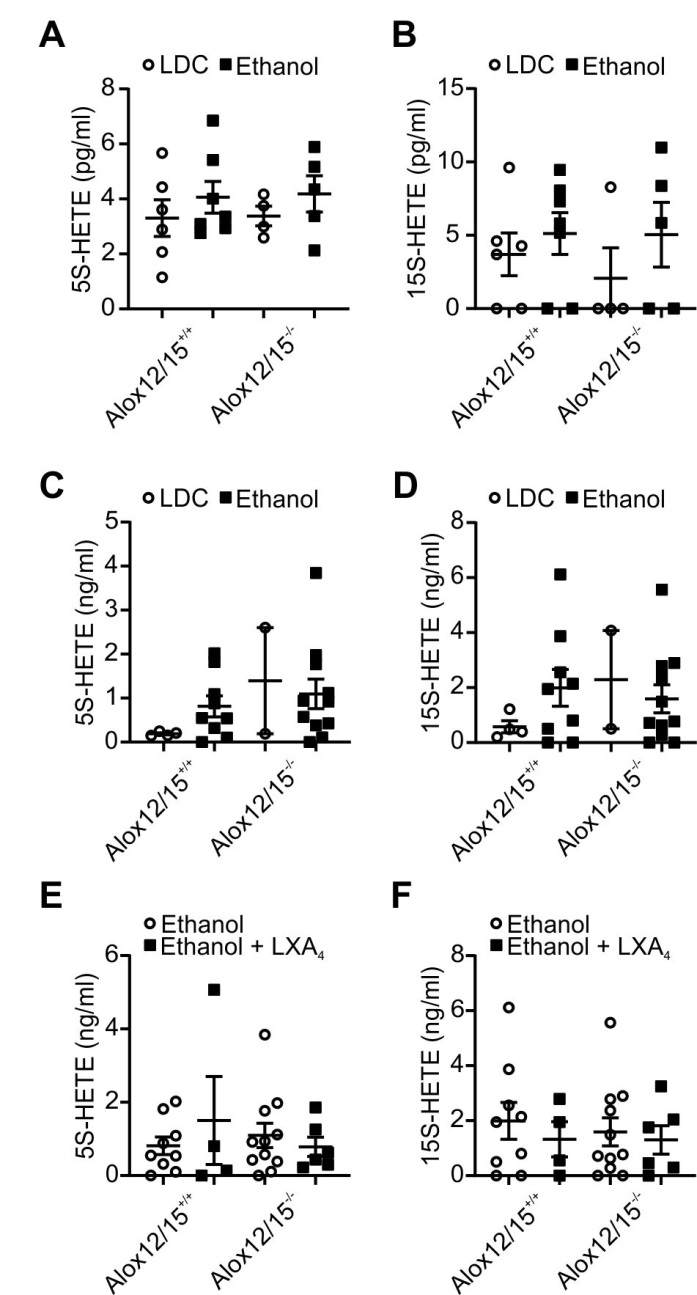


**Figure S5: Systemic and hepatic primary Alox products in AH.** (A-D) Alox12/15^+/+^ and

Alox12/15^-/-^ mice were subjected to the NIAAA (National Institute of Alcoholism and Alcohol Abuse) model of alcoholic hepatitis (AH). After caloric acclimatization for five days, mice were fed with an ethanol containing diet for 11 d compared with an isocaloric liquid diet without ethanol (Lieber-DeCarli; LDC), followed by binge-feeding with ethanol or an isocaloric control. Levels of hepatic Alox12/15 products 5S-hydroxyeicosatetraenoic acid (5S-HETE) (A), 15S-HETE (B), as well as systemic levels of 5S-HETE (C), and 15S-HETE (D) were determined by LC-MS/MS). Animal numbers were (A-B) Alox12/15^+/+^: LDC N=6, Ethanol N=7; Alox12/15^-/-^: LDC N=4, Ethanol N=5 (C-D) Alox12/15^+/+^: LDC N=4, Ethanol N=9; Alox12/15^-/-^: LDC N=2, Ethanol N=11. (E-F) Alox12/15^+/+^ and Alox12/15^-/-^ mice were fed with an ethanol containing diet for 11 d followed by binge-feeding with ethanol, with or without repetitive i.p. injections of 10 µg/kg lipoxin A_4_ (LXA_4_) at days 5, 7, 9 and 11 during chronic ethanol feeding. Systemic levels of 5S-HETE (E), and 15S-HETE (F) were determined by LC-MS/MS. Animal numbers were (E-F) Alox12/15^+/+^: Ethanol N=9, Ethanol + LXA_4_ N=4; Alox12/15^-/-^: Ethanol N=11, Ethanol + LXA_4_ N=6. Each data point corresponds to data from one individual animal. For statistical analyses, Mann-Whitney test was used.

**Figure S6**


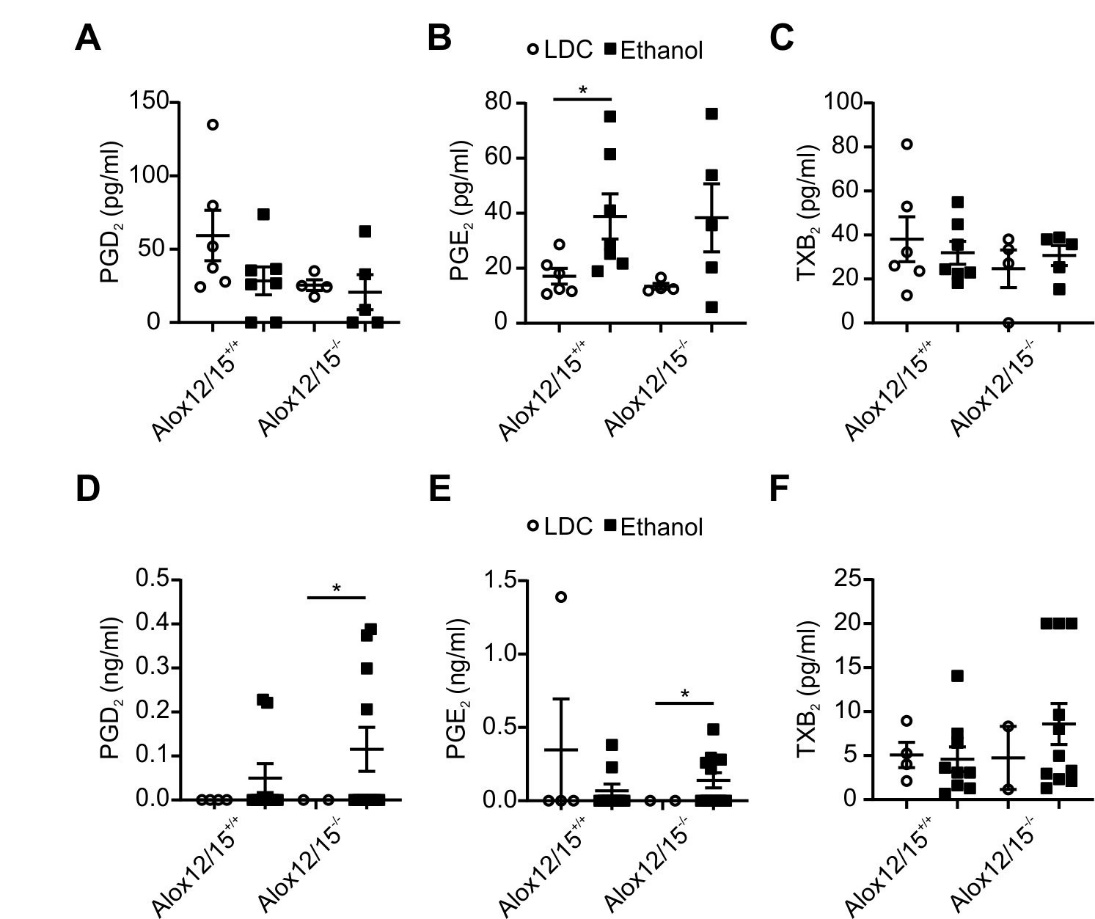


**Figure S6: Systemic and hepatic prostanoid levels in AH.** Alox12/15^+/+^ and Alox12/15^-/-^ mice were subjected to the NIAAA (National Institute of Alcoholism and Alcohol Abuse) model of alcoholic hepatitis (AH). After caloric acclimatization for five days, mice were fed with an ethanol containing diet for 11 d compared with an isocaloric liquid diet without ethanol (Lieber-DeCarli; LDC), followed by binge-feeding with ethanol or an isocaloric control. Levels of hepatic prostaglandin D_2_ (PGD_2_) (A), prostaglandin E_2_ (PGE_2_) (B), and thromboxane B_2_ (TXB_2_) (C), as well as systemic levels of PGD_2_ (D), PGE_2_ (E), and TXB_2_ (F) were determined by LC-MS/MS. Animal numbers were (A-C) Alox12/15^+/+^: LDC N=6, Ethanol N=7; Alox12/15^-/-^: LDC N=4, Ethanol N=5 (D-F) Alox12/15^+/+^: LDC N=4, Ethanol N=9; Alox12/15^-/-^: LDC N=2, Ethanol N=11. Each data point corresponds to data from one individual animal. * p ≤ 0.05. For statistical analyses Mann-Whitney test was used.

**Figure S7**


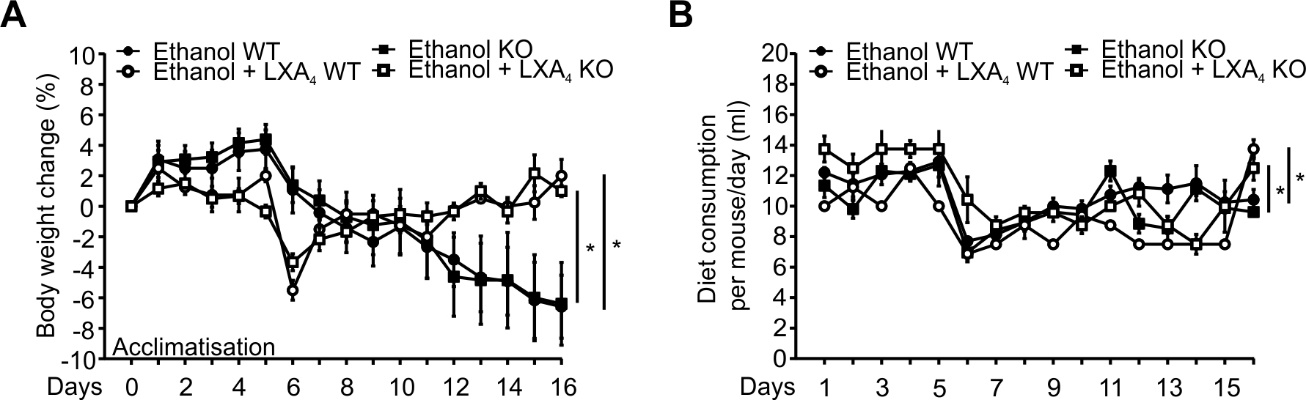


Figure S7. Impact of LXA_4_ on body weight and diet intake during AH. Alox12/15^+/+^ and Alox12/15^-/-^ mice were fed with an ethanol containing diet for 11 d followed by binge-feeding with ethanol, with or without repetitive i.p. injections of 10 µg/kg lipoxin A_4_ (LXA_4_) at days 5, 7, 9 and 11 during chronic ethanol feeding. (A) Change in body weight of Alox12/15^+/+^ (WT) and Alox12/15^-/-^ (KO) mice over time, and (B) diet intake over time are shown. Animal numbers were (A) Alox12/15^+/+^: Ethanol N=12; Alox12/15^-/-^: N=13 (B) Alox12/15^+/+^: Ethanol + LXA_4_ N=4; Alox12/15^-/-^: Ethanol + LXA_4_ N=6. Data are means ± SEM. * p ≤ 0.05. For statistical analyses of differences in daily body weight change (A), respectively diet consumption (B) between Alox12/15^+/+^ and Alox12/15^-/-^ mice after ethanol treatment with, or without LXA_4_ injections, Mann-Whitney test was used.

**Figure S8**

**Figure S8. Representative Sirius Red staining (magnification 40X) for detection of hepatic fibrosis in AH with Lipoxin A_4_ treatment.** Alox12/15^+/+^ mice fed with Ethanol without Lipoxin A_4_ i.p. injection (top left) and with Lipoxin A_4_ i.p. injection (bottom left) as well as Alox12/15^-/-^ mice fed with Ethanol without Lipoxin A_4_ i.p. injection (top right) and with Lipoxin A_4_ i.p. injection (bottom right). Fibrosis appears as red structures.

## Supplementary Tables

**Table-S1**

| **Immunophenotyping of immune cell subtypes** | | | | | |
| --- | --- | --- | --- | --- | --- |
| **Antibodies** | **Fluorochrome** | **Company** | **Antibody-ID** | **Clone** | **Species** |
| Anti-CD3 | PE-CF594 | BD Biosciences | [AB 2737959](http://antibodyregistry.org/AB_2737959) | 145-2C11 | Hamster |
| Anti-CD4 | BV711 | BD Biosciences | [AB 2737973](http://antibodyregistry.org/AB_2737973) | GK15 | Rat |
| Anti-CD8 | BV650 | Bio Legend | [AB 2738084](http://antibodyregistry.org/AB_2738084) | 53-6.7 | Rat |
| Anti-CD11b | BV605 | Bio Legend | [AB 11126744](http://antibodyregistry.org/AB_11126744) | M170 | Rat |
| Anti-CD11c | AlexaFluor700 | BD Biosciences | [AB 2737617](http://antibodyregistry.org/AB_2737617) | HL3 | Hamster |
| Anti-CD19 | APC-H7 | BD Biosciences | [AB 398483](http://antibodyregistry.org/AB_398483) | 1D3 | Rat |
| Anti-CD31 | PE-Cy7 | eBioscience | [AB 468932](http://antibodyregistry.org/AB_468932) | 390 | Rat |
| Anti-CD34 | FITC | BD Biosciences | [AB 395015](http://antibodyregistry.org/AB_395015) | RAM34 | Rat |
| Anti-CD38 | BV510 | BD Biosciences | [AB 2739886](http://antibodyregistry.org/AB_2739886) | 90/CD38 | Rat |
| Anti-CD45 | VioBlue | Miltenyi | [AB 2659925](http://antibodyregistry.org/AB_2659925) | 30F11 | Rat |
| Anti-CD90.2 | PE | Miltenyi | [AB 2659874](http://antibodyregistry.org/AB_2659874) | 30H12 | Rat |
| Anti-CD117 | APC-eFluor780 | eBioscience | [AB 469432](http://antibodyregistry.org/AB_469432) | ACK2 | Rat |
| Anti-CD138 | PE | BD Biosciences | [AB 2033998](http://antibodyregistry.org/AB_2033998) | 281-2 | Rat |
| Anti_CD326 | BV711 | BD Biosciences | [AB 2738022](http://antibodyregistry.org/AB_2738022) | G8.8 | Rat |
| Anti-GITR | FITC | Bio Legend | [AB 1089125](http://antibodyregistry.org/AB_1089125) | DTA-1 | Rat |
| Anti-SiglecH | FITC | Bio Legend | [AB 1227760](http://antibodyregistry.org/AB_1227760) | 551 | Rat |
| Anti-MerTK | PE-Cy7 | eBioscience | [AB 2572622](http://antibodyregistry.org/AB_2572622) | DS5MMER | Rat |
| Anti-F4/80 | PE-Cy7 | Bio Legend | [AB 893498](http://antibodyregistry.org/AB_893498) | BM8 | Rat |
| Anti-γδTCR | APC | Thermo Fisher | [AB 842757](http://antibodyregistry.org/AB_842757) | GL3 | Hamster |
| Anti-HLA-DR | APC | Miltenyi | [AB 244259](http://antibodyregistry.org/AB_244259) | M5/114.15.2 | Rat |
| Anti-IgM | BV650 | BD Biosciences | [AB 2741427](http://antibodyregistry.org/AB_2741427) | Il/41 | Rat |
| Anti-Ly-6C | PerCP-Cy5.5 | BD Biosciences | [AB 2737749](http://antibodyregistry.org/AB_2737749) | AL-21 | Rat |
| Anti-Ly-6G | APC-Cy7 | Bio Legend | [AB 1877163](http://antibodyregistry.org/AB_1877163) | 1A8 | Rat |
| Anti-NK1.1 | AlexaFluor 700 | BD Biosciences | [AB 2728688](http://antibodyregistry.org/AB_2728688) | PK136 | Mouse |

**Table-S1: Antibody panel for immunophenotyping of immune cell subtypes.**

Antibody-identification numbers were collected from antibodyregistry.org.
